# Fore-Warned Is Fore-Armed: Effect of Musculoskeletal Disorders on Sickness Absenteeism

**DOI:** 10.7759/cureus.30481

**Published:** 2022-10-19

**Authors:** Varsha R Mokhasi

**Affiliations:** 1 Department of Community Medicine, Sri Siddhartha Medical College, Tumkur, IND

**Keywords:** occupational hazards, garment workers, predictive factors, sickness absence, musculoskeletal disorders

## Abstract

Background

Musculoskeletal disorders (MSDs) are one of the most common occupational diseases. MSDs cause an economic burden as they lead to disability, absenteeism, and reduced productivity. Although many risk factors have been associated with the incidence of MSDs, little is known about the predictive factors for the length of MSD-related absenteeism. The study aimed to estimate the prevalence of MSDs among garment factory workers, determine the predictive factors for length of sickness absence, and evaluate the association of these factors with time.

Methodology

This prospective, observational, community-based study was conducted among garment factory workers. A total of 430 participants were included in the study by multistage sampling to assess the prevalence and factors causing MSDs, followed by a one-year follow-up to analyze the MSD-associated predictors for sickness absence. The data were analyzed using SPSS version 22 (IBM Corp., Armonk, NY, USA). The chi-square test and logistic regression were used to test significance, and Cox regression was used to determine the predictive factors for sickness absence.

Results

The mean age of the participants was 29.36 years. The 12-month prevalence showed that the most affected region was the lower back (70%), followed by the upper back (40%), with some workers experiencing both. There was a significant relation between MSDs and higher age, low work experience, being overweight, and long work hours (p < 0.05). According to the multivariate analysis, the perceived pain (hazards ratio (HR) = 1.14), perceived physical workload (HR = 1.14), and visiting a specialist 12 months prior to the current absence (HR = 1.68) were significantly associated with a longer sickness absence.

Conclusions

The lower and upper back were most commonly affected. The predictors for absence revealed that physical work overload and pain perception had a predominant role in the duration of absence. Hence, an ergonomically acceptable workplace with considerate rest periods will help enhance productivity.

## Introduction

Musculoskeletal conditions encompass around 150 diagnoses that affect the locomotor system including the muscles, joints, bones, and related tissues such as the tendons and ligaments, as enumerated in the International Classification of Diseases, Tenth Revision (ICD-10). These conditions vary from sudden and short-lived, such as sprains, strains, and fractures, to long-lasting conditions associated with ongoing pain and disability.

Occupation-related musculoskeletal disorders (MSDs) are often associated with persistent pain which may lead to limitations in mobility, work proficiency, and functional abilities, thus diminishing the ability to work with associated impacts on mental well-being. Broadly, MSDs influence productivity and quality of work. The most common and disabling musculoskeletal conditions are osteoarthritis, back and neck pain, and systemic inflammatory conditions such as rheumatoid arthritis [1].

The textile industry in India is the second largest manufacturer and exporter globally, succeeding China as per the 2019 reports. It contributes 2% of the gross domestic product (GDP) and 15% of India’s export earnings. It is one of the largest sources generating jobs in the country, with more than 46 million people employed directly, which includes a large number of women and the rural population [2].

Kolar is one of the salient junctions identified under the Bangalore-Chennai Industrial Corridor, which contributes 1.89% to Karnataka’s GDP from its industries. There are around 13 ready-made garment factories providing employment to 8,065 workers.

Textile industry workers mostly report pain in the neck, back, and shoulders. This may be attributed to the nature of the work which involves prolonged sitting, repetitive work, vibration, incorrect work postures, and extensive workload. These factors lead to increased pressure on ligaments, muscles, and other soft tissues, causing pain [3].

There are numerous established risk factors known to cause MSDs. However, considerably less is known whether these predictors also act as contributors to extended sickness absence (two to six weeks) because of MSDs. In this study, we were intrigued about the length of sickness absence as an estimate of the chronic persistence of musculoskeletal conditions [4,5]. The main factors that are usually associated with longer sickness absence are age, perception of physical workload, and poor general health [6,7].

This study aimed to estimate the prevalence of MSDs and the factors causing MSDs among women employed in garment factories. The second goal was to determine the predictive factors for the length of sickness absence due to MSDs and evaluate the association of these factors with time.

## Materials and methods

Kolar district has five government-registered industrial areas. This prospective observational study was conducted in Bellambari, an industrial area in Kolar taluk, which has a ready-made garments factory with 800 employees from January 2019 to February 2020. A sample size of 430 was estimated based on a 36% prevalence of MSDs as per the Indian Council of Medical Research (ICMR) musculoskeletal conditions epidemiology, with a 95% confidence interval (CI), absolute precision of 5%, and a 20% dropout rate using n Master [8].

**Figure 1:**
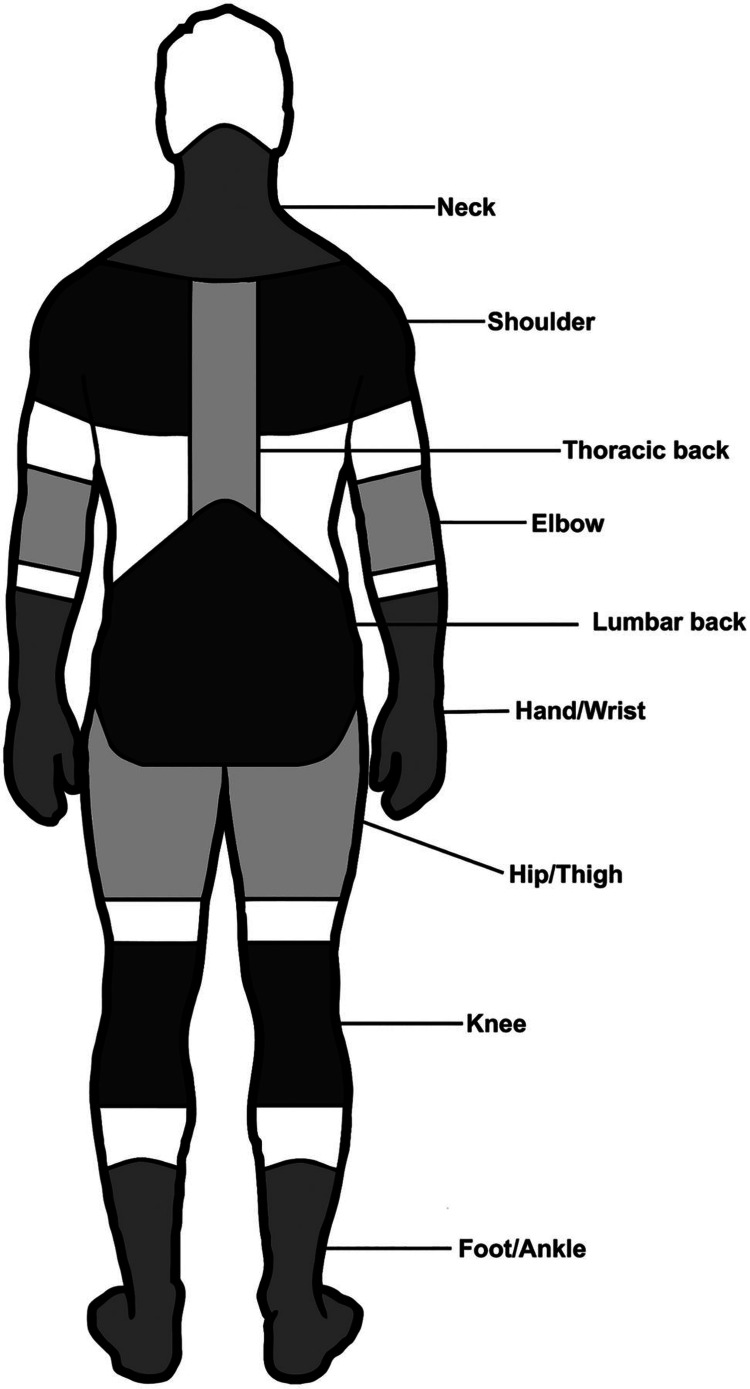
Nordic nine-point questionnaire mannequin illustrating the defined anatomical areas for assessing musculoskeletal pain. Monnier A, Larsson H, Djupsjobacka M. Musculoskeletal pain and limitations in work ability in Swedish marines: a cross-sectional survey of prevalence and associated factors. BMJ Open. 2015;5:43-79 [10]. This article is available under the Creative Commons CC-BY-NC license and permits non-commercial use, distribution, and reproduction in any medium provided the original work is properly cited.

Multistage sampling was employed in the study. Out of the three garment factories in our field practice area, the Bellambari area was selected. Among the workers employed, 430 workers were selected by simple random sampling, which comprised those with at least one year of work experience. The team comprised interns, consultants in community medicine, and Medico social workers. The inclusion criteria were age >18 years and those who had at least one year of working experience. Pregnant women and disabled individuals were not included in the study. The interns were trained in data collection as per the Nordic questionnaire: nine pain points along with section B with the assessment of reasons for sickness absenteeism. The Nordic questionnaire has a general section identifying nine areas of the body causing musculoskeletal problems as depicted in Figure 1. In this questionnaire, the respondents are asked if they have had any musculoskeletal pain in any of the areas in the last 12 months and the last seven days [9].

In this study, an attempt was made for a different approach in analyzing the factors predicting the length of sickness absenteeism due to MSDs. After collecting the baseline data about the prevalence and factors affecting MSDs, a longitudinal prospective study was conducted for the next 12 months. Workers taking two to six weeks of leave were included and followed up for a year to determine the predictive factors for the length of sickness absence due to MSDs. This selection was made based on the confirmatory clinical diagnosis as given by a trained medical officer placed there as well as from the details obtained from the registry maintained at the factory. Information was collected by trained researchers using a pre-validated, structured questionnaire over the telephone for workers on MSD-related sick leave.

A health camp was conducted comprising physicians, obstetricians, and orthopedics to address MSDs and other health concerns, and counseling was done regarding ergonomics and adoption of proper posture to reduce the suffering caused by MSDs, thus helping to reduce the incidence of sickness absenteeism and improve the production in the factory.

The perception of physical workload was also measured using a rating system on a 10-point scale [11]. To analyze work-related psychosocial factors, the Job Content Questionnaire was employed [12]. The pain severity as perceived by workers was assessed using a 10-point scale [13]. The functional disability associated with the illness was evaluated using the Roland-Morris Disability Questionnaire [14].

Ethical approval was obtained from the Ethical Review Committee of Sri Devaraj Urs Academy of Higher Education and Research. Permission to recruit workers was obtained from the respective garment factories. Written informed consent was obtained from each participant. Interviews were conducted at a time and place inside the factory premises which was convenient for the participants to collect baseline data.

The data were analyzed using SPSS version 22 (IBM Corp., Armonk, NY, USA). The quantitative measures are presented by mean and standard deviation and qualitative variables as proportions. The chi-square and logistic regression tests were used to test significance. P-values of ≤0.05 were considered statistically significant. The Cox hazards regression test was used to determine the predictive factors for the length of sickness absence.

## Results

The demographic characteristics of the participants are presented in Table 1. All respondents were women with a mean age of 29.36 years (SD = 6.96). The majority of the participants either had no formal schooling (30.7%) or only primary education (43%). Among the total participants, 53.7% were sewing operators and 93.2% were doing overtime work (>8 hours). The majority (286, 66.5%) were earning <7,000 INR (85 US dollars) including overtime payments.

**Table 1:** Demographic characteristics of the participants.

Characteristics		Participants (n = 430)
Age	Mean ± SD	29.36 ± 6.96
	<30	277 (64.4%)
	30–40	133 (30.9%)
	>40	20 (4.7%)
Education	Illiterate	132 (30.7%)
	Primary school	185 (43%)
	High school	92 (21.4%)
	Pre-university	21 (4.9%)
Current occupation	Sewing operator	231 (53.7%)
	Cutting section	99 (23%)
	Warehouse	49 (11.4%)
	Iron section	51 (11.9%)
Daily working hours	8 hours	29 (6.74%)
	>8 hours	401 (93.2%)
Monthly income (INR)	Mean ± SD	7,658 ± 2,064
	<7,000	286 (66.5%)
	7,000–14,000	144 (33.5%)
BMI (kg/m^2^)	Mean ± SD	23.4 ± 2.7
	Underweight	82 (19.06%)
	Normal	186 (43.2%)
	Overweight and obese	162 (37.6%)

Around 88.6% (381) of the respondents were below the poverty line. The mean weight, height, and body mass index (BMI) were 53.9 kg (SD = 11.56), 1.55 m (SD = 0.068), and 22.34 kg/m^2^ (SD = 4.51), respectively, with 19.1% of the participants being underweight.

Our study revealed that the one-week prevalence of MSDs was the highest in the lower back (60%), followed by the upper back (30%), foot/ankle (27.9%), and wrist (24%) (Table 2). This finding was highly statistically significant.

**Table 2:** Distribution of the study participants according to the standardized Nordic questionnaire. MSDs: musculoskeletal disorders

Body parts affected by MSDs	In the past 12 months		In the past 7 days		Disabling attack		χ^2^ value	P-value
	N	%	N	%	N	%		
Neck								
Yes	109	25.3	95	22.1	52	12.1	25.7996	<0.0001
No	321	74.7	335	77.9	378	87.9		
Shoulder								
Yes	103	24	95	22.1	43	10	32.4938	<0.0001
No	327	76	335	77.9	387	90		
Elbow								
Yes	60	14	43	10	30	7	11.3843	0.0033
No	370	86	387	90	400	93		
Wrist								
Yes	149	34.7	103	24	39	9.1	81.2582	<0.0001
No	281	65.3	327	76	391	90.9		
Upper back								
Yes	172	40	129	30	74	17.2	54.431	<0.0001
No	258	60	301	70	356	82.8		
Lower back								
Yes	303	70.5	258	60	107	24.9	196.34	<0.0001
No	127	29.5	172	40	323	75.1		
Hip								
Yes	60	14	53	12.3	21	4.9	21.602	<0.0002
No	370	86	377	87.7	409	95.1		
Knees								
Yes	73	17	43	10	43	10	12.91	0.0015
No	357	83	387	90	387	90		
Foot/Ankle								
Yes	129	30	120	27.9	86	20	12.44	0.0019
No	301	70	310	72.1	344	80		

MSD prevalence during the last 12 months also showed a similar pattern in the frequency of occurrence, with the lower back (70%) being most prevalent, followed by the upper back (40%), wrist (34.7%), and foot/ankle (30%), which was also statistically significant.

The most disabling MSDs affecting participants was the upper back (17.2%), followed by the lower back (24.9%), foot (20%), and neck (12.1%).

**Figure 2:**
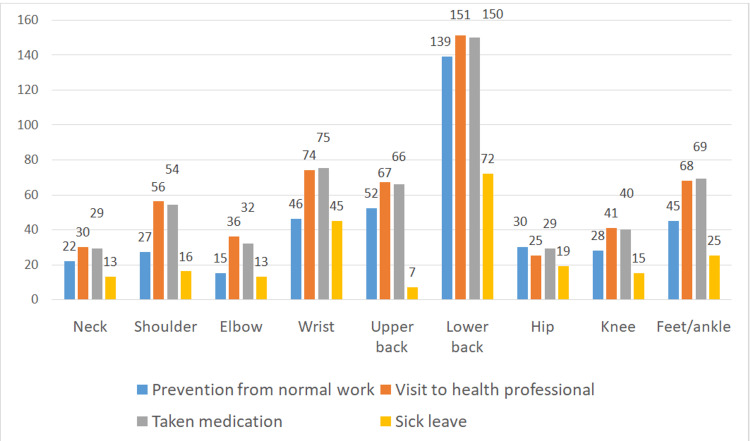
Musculoskeletal pain-related effects on different body regions.

Due to lower back pain, a significant number of both males and females suffered from prevention of normal work activities, had to visit health professionals, took medications, and went on sick leaves (Figure 2). The factors associated with the one-year prevalence of MSDs in the different regions of the body by age, job experience, BMI, and daily working hours are depicted in Table 3. There was a significant relationship between MSDs in wrist (p = 0.05), lower back (p = 0.02), knee (p = 0.02), and ankle (p = 0.012) with age; upper back (p = 0.01), elbows (p = 0.01), and hips (p =0.05) with job experience; hips (p = 0.01), knee (p = 0.03), and ankle (p = 0.05) with BMI; and wrist (p = 0.001) and lower back (p = 0.001) with daily working hours.

**Table 3:** Association of factors with the one-year prevalence of musculoskeletal disorders in different regions of the body (n = 430).

Factors	Neck	Shoulder	Upper Back	Elbow	Wrist/Hand	Lower back	Hip	Knee	Ankle/Feet
	χ^2^ (p)	χ^2^ (p)	χ^2^ (p)	χ^2^ (p)	χ^2^ (p)	χ^2^ (p)	χ^2^ (p)	χ^2^ (p)	χ^2^ (p)
Age (years)									
<30	2.38 (0.3)	0.181 (0.9)	2.62 (0.26)	2.44 (0.29)	5.96 (0.05)	7.24 (0.02)	3.1 (0.2)	7.43 (0.02)	8.8 (0.01)
30–40									
>40									
Job experience (years)									
<5	1.11 (0.5)	2.86 (0.23)	13.1 (0.01)	13.9 (0.01)	2.06 (0.3)	2.77 (0.25)	5.76 (0.05)	4.87 (0.8)	5.6 (0.6)
5–10									
>10									
Body mass index (kg/m^2^)									
Underweight	0.86 (0.6)	0.15 (0.96)	0.21 (0.91)	0.37 (0.82)	0.16 (0.9)	0.45 (0.79)	7.46 (0.01)	6.85 (0.03)	5.72 (0.05)
Normal weight									
Overweight and obese									
Daily working hours									
8 hours	0.75 (0.4)	1.39 (0.23)	0.23 (0.62)	3.98 (0.04)	0.001 (0.9)	0.001 (0.94)	0.33 (0.5)	0.46 (0.4)	0.54 (0.46)
>8 hours									

Of the 242 participants, 232 (95.8%) returned to work fully within the 12-month follow-up period. Table 4 presents the sickness absence duration, in general, and stratified by lower and upper back, neck, hip, knee, and foot. The median sickness absence duration for the entire population was 89 days, ranging from 12 to 180 days (Table 4).

**Table 4:** Stratification of absence due to musculoskeletal disorders by affected body parts.

Reason for sickness absence	Number (%)
Total	242
Neck	24 (9.9%)
Upper back	42 (17.4%)
Lower back	133 (54.9%)
Hip	16 (6.6%)
Knee	20 (8.3%)
Foot/Ankle	7 (2.9%)

Table 5 presents the predictive factors for the duration of sickness absence due to MSDs in both the univariate and multivariate analyses. In the univariate analysis, the presence of poor interpersonal relationships at the workplace, perceived pain, high perceived physical workload, functional disability, and the need for a doctor’s visit in 12 months prior to the present sickness absence were statistically significantly associated with longer sickness absence.

**Table 5:** Univariate and multivariate analysis for predictive factors for the length of sickness absenteeism due to musculoskeletal disorders among participants on leave for two to six weeks. HR: hazard ratio; CI: confidence interval

	Musculoskeletal disorders			
	Univariate analysis		Multivariate analysis	
Predictive factors	HR	CI (95%)	HR	CI (95%)
Age	0.98	0.75-1.29	1.21	0.89-1.65
Physical factors (workload perceived)	1.10	1.00-1.23	1.14	1.03-1.26
Psychosocial factors: interpersonal relation (work)	0.90	0.82-1.00	0.92	0.83-1.04
Pain severity as perceived	1.31	1.00-1.74	1.14	0.83-1.54
Functional disability	1.05	0.99-1.14	0.98	0.89-1.07
History of sick leave (need for a doctor’s visit in 12 months)	1.43	1.04-1.95	1.68	1.17-2.40

In the multivariate analysis, only the perceived pain, perceived physical workload, and visiting a specialist 12 months prior to the current sickness absence period were statistically significantly associated with longer sickness absence. The hazard ratio of older age increased to 21% in the multivariate analysis.

In the univariate analysis of MSDs, perceived physical workload and functional disability showed a statistically significant association with time. Workers with a high perceived physical workload returned to work increasingly slower over time, whereas workers with a high functional disability returned to work increasingly faster over time. Although in the multivariate analysis the effect of both interactions remained the same, only perceived physical workload was statistically significant.

## Discussion

This is one of the distinctive prospective studies conducted in South India to assess not only the prevalence of MSDs but also analyze the predicting factors contributing to workplace absence due to MSDs.

The participants employed in the garment factory included in our study were women and were in the 18-30-year age group, indicating that more women belonging to the class of early working age population are employed in garment factories than men. This can be explained by the fact that female school drop-out rates are still high, and females seek garments as a source of economic support [15]. This observation is consistent with another study in the south Indian state of Tamil Nadu by Sreesupria et al. [16].

We also found that most workers had received primary education which is comparable with the National Statistical Office (NSO) data of the Periodic Survey by Labour Force (58.4%) [17].

There is an increasing demand for products from the ready-made garment sector, especially of export quality, in our country and more so in the industrial area of our district. This leads to an escalated demand and the direct impingement of this is over-time working [18]. This results in poor health outcomes among workers [19].

Another important finding was that 37.6% of the respondents were either overweight or obese, which can be explained by the evidence that 53.7% of the participants were workers from the sewing section. Workers in this section sit and work for prolonged hours. This was contradictory to the observation made by Vidusha et al. where only 20% of the workers belonged to the overweight and obese category, which might be due to the inclusion of more workers who had a standing job in the study (61.2%) [20]. The mean BMI (23.4 ± 2.7) of our study is comparable with a study by Hossain et al. where the majority of the workers were sewing machine operators [21].

The occurrence of low back pain (60%) was of greater preponderance than the other nine body parts assessed as per the Nordic questionnaire, which is consistent with the findings of researchers worldwide [22,23]. Another study at multiple garment factories in Jaipur, India, also reported similar findings [24]. Some prior studies by researchers from Kolkata and Bangladesh also indicated that ready-made garment workers suffer from neck and back pain the most [25,26].

It was noticed that during working hours, aside from the lunch break of 30 minutes, there was no rest period provided to workers. There is proof to support the fact that short breaks during working hours help workers relieve their fatigue and minimize the risk of developing low backache, thus improving productivity and quality of work [27].

There was statistical significance in relation to the one-year prevalence of MSDs and some of the crucial sociodemographic factors such as age, BMI, job experience, and daily working hours. A few of the earlier studies have also shown similar findings [28,29].

MSDs and sickness absenteeism

The main predictors found to be connected with the long duration of sickness absenteeism were poor interpersonal relationships at the workplace, perceived pain, high perceived physical workload, functional disability, and the need for a medical visit 12 months prior. Employees with a higher perceived physical load of work showed a more leisurely pace of return to work than others.

Predictive factors and length of sickness absenteeism

The findings of our study regarding the importance of perceived pain are consistent with the studies conducted among factory employees on sickness absenteeism. Even though there are not many studies in our country that concentrate on the importance of predictive factors for the length of absence from work, some studies have shown that physical workload is a significant factor in the length of absence from work [30].

The effect of pain intensity on the duration of sickness absence remained constant and was independent of adjustment for perceived functional disability.

Even though disability affecting function is an important predictive factor for an extended absence from work, the results showed that greater initial intensity of pain overrules functional disability.

The state of panic related to the pain leading to an inability to perform or chances of worsening pain is proven to be a deciding factor in the length of absence. Another suggestion is that such terrorizing thoughts and their effects can be minimized by changing their beliefs and improving their capability to perform physical work effectively.

Having said that, not just positive psychological reinforcement but also effective programs at work such as encouraging and teaching proven muscular exercises to avoid outcomes of MSDs are crucial. This can only be achieved by creating a work program that encompasses an ergonomically suited work environment, resting hours, health education about work postures, and healthy interpersonal relationships.

We would also like to add that the further continuation of the study will be in educating both the employers and workers in line with the above observed corrective measures to achieve a healthy work environment and increase productivity as its collateral outcome.

## Conclusions

As disclosed by the 12-month prevalence, the lower and upper back were the most commonly affected anatomical regions. It was also observed that lower work experience, longer working hours, and higher BMI played a major role in causing MSDs. The predictors for absence due to MSDs revealed that along with physical work overload, pain perception had a predominant role in the duration of absence. Hence, a healthy work environment that is ergonomically acceptable with considerate rest periods will help enhance productivity. This can be achieved by interventions tailor-made to fulfill the needs of workers in terms of ergonomics such as postures of sitting or standing and hand movements, exercises to strengthen the musculature, psychosocial well-being, and, finally, yet importantly, to strike an exact work-rest balance.

Therefore, the recommendation of low-cost solutions is essential for providing adequate health education to workers regarding ergonomics with respect to work posture, duration of repetitive work, and mandatory resting after four to five hours of continuous work. These should be made an integral part of the work policy and should be implemented effectively.
